# Characteristics and Outcomes of Women With COVID-19 Giving Birth at US Academic Centers During the COVID-19 Pandemic

**DOI:** 10.1001/jamanetworkopen.2021.20456

**Published:** 2021-08-11

**Authors:** Justine Chinn, Shaina Sedighim, Katharine A. Kirby, Samuel Hohmann, Afshan B. Hameed, Jennifer Jolley, Ninh T. Nguyen

**Affiliations:** 1 University of California, Irvine Medical Center, Orange; 2 Vizient, Centers for Advanced Analytics, Chicago, Illinois

## Abstract

**Importance:**

Prior studies on COVID-19 and pregnancy have reported higher rates of cesarean delivery and preterm birth and increased morbidity and mortality. Additional data encompassing a longer time period are needed.

**Objective:**

To examine characteristics and outcomes of a large US cohort of women who underwent childbirth with vs without COVID-19.

**Design, Setting, and Participants:**

This cohort study compared characteristics and outcomes of women (age ≥18 years) who underwent childbirth with vs without COVID-19 between March 1, 2020, and February 28, 2021, at 499 US academic medical centers or community affiliates. Follow-up was limited to in-hospital course and discharge destination. Childbirth was defined by clinical classification software procedural codes of 134-137. A diagnosis of COVID-19 was identified using *International Statistical Classification of Diseases and Related Health Problems, Tenth Revision* (*ICD-10*) diagnosis of U07.1. Data were analyzed from April 1 to April 30, 2021.

**Exposures:**

The presence of a COVID-19 diagnosis using *ICD-10*.

**Main Outcomes and Measures:**

Analyses compared demographic characteristics, gestational age, and comorbidities. The primary outcome was in-hospital mortality. Secondary outcomes included hospital length of stay, intensive care unit (ICU) admission, mechanical ventilation, and discharge status. Continuous variables were analyzed using *t* test, and categorical variables were analyzed using χ^2^.

**Results:**

Among 869 079 women, 18 715 (2.2%) had COVID-19, and 850 364 (97.8%) did not. Most women were aged 18 to 30 years (11 550 women with COVID-19 [61.7%]; 447 534 women without COVID-19 [52.6%]) and were White (8060 White women [43.1%] in the COVID-19 cohort; 499 501 White women (58.7%) in the non–COVID-19 cohort). There was no significant increase in cesarean delivery among women with COVID-19 (6088 women [32.5%] vs 273 810 women [32.3%]; *P* = .57). Women with COVID-19 were more likely to have preterm birth (3072 women [16.4%] vs 97 967 women [11.5%]; *P* < .001). Women giving birth with COVID-19, compared with women without COVID-19, had significantly higher rates of ICU admission (977 women [5.2%] vs 7943 women [0.9%]; odds ratio [OR], 5.84 [95% CI, 5.46-6.25]; *P* < .001), respiratory intubation and mechanical ventilation (275 women [1.5%] vs 884 women [0.1%]; OR, 14.33 [95% CI, 12.50-16.42]; *P* < .001), and in-hospital mortality (24 women [0.1%] vs 71 [<0.01%]; OR, 15.38 [95% CI, 9.68-24.43]; *P* < .001).

**Conclusions and Relevance:**

This retrospective cohort study found that women with COVID-19 giving birth had higher rates of mortality, intubation, ICU admission, and preterm birth than women without COVID-19.

## Key Points

**Question:**

What are the characteristics and outcomes associated with giving birth with COVID-19 over the first year of the pandemic in the US?

**Findings:**

This cohort study examines 869 079 adult women, including 18 715 women with COVID-19, who underwent childbirth at 499 US medical centers between March 1, 2020, and February 28, 2021. Women with COVID-19 had increased mortality, need for intubation and ventilation, and intensive care unit admission.

**Meaning:**

These findings suggest that COVID-19 was associated with increased risk of morbidity and mortality for women giving birth.

## Teaser

This cohort study examines morbidity and mortality outcomes among US women giving birth with COVID-19.

## Introduction

Although there has been extensive research on COVID-19 since its initial emergence, the broad range of perinatal COVID-19 outcomes continues to emerge.^1^ A study by Jering and colleagues^2^ reported the largest US cohort, to date, including 6380 women with COVID-19 giving birth, and found that the absolute death rates in women with COVID-19 were considerably higher than in women without COVID-19. They also found higher rates of preterm birth, preeclampsia, and thrombotic events.^2^ Prior studies on the effects of COVID-19 on pregnancy have reported higher rates of cesarean delivery, preterm birth, and maternal morbidity and mortality index scores.^3,4,5^ As the COVID-19 pandemic has progressed, mortality and outcomes in the general population have improved.^6^ The aim of this study was to examine the characteristics and outcomes of a large US cohort of women who underwent childbirth with vs without COVID-19 during the first year of the pandemic, examining a larger single data set cohort of women with COVID-19 over a longer period than prior studies, to our knowledge.

## Methods

This cohort study was determined exempt from review and informed consent by the Institutional Review Board of the University of California, Irvine, owing to lack of patient-identifying information. Approval for the use of the data was obtained from Vizient. This study followed the Strengthening the Reporting of Observational Studies in Epidemiology (STROBE) reporting guideline.

Using the Vizient Clinical Data Base/Resource Manager (CDB/RM), we compared characteristics and outcomes of women (age ≥18 years) who underwent childbirth with vs without COVID-19 between March 1, 2020, and February 28, 2021. The CDB/RM is an administrative, clinical, and financial database of more than 650 academic centers and their community affiliates. Data represent clinical information submitted by 97% of academic medical centers across the US, representing a broad range of patients across geographic zones. Childbirth was identified based on clinical classification software procedural codes of 134 for cesarean delivery; 135 for forceps, vacuum, or breech delivery; 136 for artificial rupture of membrane to assist delivery; and 137 for other procedures to assist delivery. A diagnosis of COVID-19 was identified using *International Statistical Classification of Diseases and Related Health Problems, Tenth Revision* (*ICD-10*)^7^ diagnosis of U07.1. Women who received a diagnosis of COVID-19 during the same admission in which they underwent childbirth were included. Demographic characteristics, gestational age, and comorbidities were compared between groups. Race and ethnicity were self-reported; these data were examined because research has found that individuals from racial and ethnic minority groups are disproportionally affected by COVID-19 compared with White individuals. The primary outcome was in-hospital mortality. Discharge disposition and in-hospital death were reported in all patients. Secondary outcomes included hospital length of stay (LOS), rates of intensive care unit (ICU) admission and mechanical ventilation, and discharge status.

### Statistical Analysis

Comparisons were performed using *t* test for continuous variables and χ^2^ analysis for categorical variables. Odds ratios (ORs) were calculated to determine the odds of mortality associated with COVID-19 for each gestational age group. Analyses were performed using Stata statistical software version 16 (StataCorp). *P* values were 2-tailed, and significance was set at *P* < .05. Data were analyzed from April 1 to April 30, 2021.

## Results

Among 869 079 women who gave birth at 499 US medical centers, 18 715 women (2.2%) had COVID-19 and 850 364 women (97.8%) did not. Most women in both cohorts were aged 18 to 30 years (11 550 women with COVID-19 [61.7%]; 447 534 women without COVID-19 [52.6%]), and 8060 women (43.1%) were White in the COVID-19 cohort, compared with 499 501 women (58.7%) who were White in the non–COVID-19 cohort (Table 1). Women with COVID-19, compared with women giving birth without COVID-19, were more likely to be Hispanic (8132 women [43.5%] vs 189 725 women [22.3%]) and/or Black (3792 women [20.3%] vs 153 783 women [18.1%]). The most common comorbidities for women giving birth with COVID-19 included obesity (3956 women [21.1%]), anemia (2310 women [12.3%]), and chronic pulmonary disease (1437 women [7.7%]). There was no significant difference in the number of cesarean deliveries performed (6088 women with COVID-19 [32.5%] vs 273 810 women without COVID-19 [32.2%]; *P* = .57). The median (range) LOS was 2 (1-211) days for women with COVID-19 and 2 (1-370) days for women without COVID-19 (*P* < .001).

**Table 1.** Demographic and Clinical Characteristics of Hospitalized Women Undergoing Childbirth With and Without COVID-19

Characteristic	Women, No. (%)		*P* value
	With COVID-19 (n = 18 715)	Without COVID-19 (n = 850 364)	
Age, y			
18-30	11 550 (61.7)	447 534 (52.6)	<.001
31-50	7165 (38.3)	403 657 (47.5)	
>50	NA^a^	173 (0.02)	
Race			
White	8060(43.1)	499 501 (58.7)	<.001
Black	3792 (20.3)	153 783 (18.1)	
Asian	659 (3.5)	49 638 (5.8)	
Unavailable	6208 (33.2)	147 445 (17.4)	
Ethnicity			
Hispanic origin	8132 (43.5)	189 725 (22.3)	<.001
Preexisting comorbidities			
Obesity	3956 (21.1)	141 486 (16.7)	<.001
Anemia	2310 (12.3)	86 890 (10.3)	<.001
Chronic pulmonary disease	1437 (7.7)	69 394 (8.2)	<.001
Drug abuse	469 (2.5)	25 388 (2.9)	<.001
Hypertension	750 (4.0)	28 068 (3.3)	<.001
Diabetes	426 (2.3)	13 737 (1.6)	<.001
Coagulation deficiency	949 (5.1)	28 702 (3.4)	<.001
Depression	972 (5.2)	56 430 (6.6)	<.001
Delivery			
Cesarean	6088 (32.5)	273 810 (32.2)	.57
Vaginal	12 627(67.5)	576 554 (67.8)	

^a^ Owing to privacy concerns, groups with fewer than 10 patients are suppressed.

Women giving birth with COVID-19, compared with those without COVID-19, had significantly higher rates of ICU admission (977 women [5.2%] vs 7943 women [0.9%]; OR, 5.84 [95% CI, 5.46-6.25]; *P*  < .001), respiratory intubation and mechanical ventilation (275 women [1.5%] vs 884 women [0.1%]; OR, 14.33 [95% CI, 12.50-16.42]; *P*  < .001), and in-hospital mortality (24 women [0.1%] vs 71 women [<0.01%]; OR, 15.38 [95% CI, 9.68-24.43]; *P* < .001) (Table 2).

**Table 2.** Clinical Outcomes of Hospitalized Women Undergoing Childbirth With and Without COVID-19

	No. (%)		Odds ratio (95% CI)	*P* value
	With COVID-19 (n = 18 715)	Without COVID-19 (n = 850 364)		
In-hospital mortality	24 (0.1)	71 (<0.01)	15.38 (9.68-24.43)	<.001
Median length of hospital stay, median (range), d	2 (1-211)	2 (1-370)	NA	<.001
ICU admission	977 (5.2)	7943 (0.9)	5.84 (5.46-6.25)	<.001
Respiratory failure and mechanical ventilation	275 (1.5)	884 (0.1)	14.33 (12.50-16.42)	<.001
Discharge status				
Home	18 477 (98.7)	845 240 (99.4)	0.47 (0.41-0.54)	<.001
Skilled nursing facility or other inpatient care	104 (0.6)	1752 (0.2)	2.71 (2.22-3.30)	<.001
Left against medical advice	102 (0.6)	1930 (0.2)	2.41 (1.97-2.94)	<.001
Died	24 (0.1)	71 (<0.01)	15.38 (9.68-24.43)	<.001

Abbreviations: ICU, intensive care unit; NA, not available.

Additionally, women with COVID-19, compared with women without COVID-19, were more likely to have a preterm delivery of less than 37 weeks (3072 women [16.4%] vs 97 967 women [11.5%]; *P* < .001). A total of 779 women with COVID-19 (4.2%) delivered at less than 32 weeks, compared with 22 355 women without COVID-19 (2.6%) (Table 3).

**Table 3.** Gestational Age and Associated Mortality of Hospitalized Women Undergoing Childbirth With and Without COVID-19

Gestational age	Women, No. (%)		Odds ratio (95% CI)	*P* value
	With COVID-19 (n = 18 715)	Without COVID-19 (n = 850 364)		
<28 wk (extremely preterm)	380 (2.0)	12 265 (1.4)	9.22 (3.70-23.01)	<.001
Mortality	NA^a^	21 (0.2)	NA	NA
28-31 wk (very preterm)	399 (2.1)	10 090 (1.2)	12.64 (3.79-42.24)	<.001
Mortality	NA^a^	NA^a^	NA	NA
32-36 wk (moderate to late preterm)	2293 (12.3)	75 612 (8.9)	22.83 (9.74-53.49)	<.001
Mortality	NA^a^	13 (<0.1)	NA	NA
≥37 wk	15 523 (82.9)	745 581(87.7)	0.44 (3.97-27.47)	<.001
Mortality	NA^a^	23 (≤0.1)	NA	NA
Unknown	120 (0.64)	6816 (2.2)	NA^b^	NA

Abbreviation: NA, not applicable.

^a^ Owing to privacy concerns, groups with fewer than 10 patients are suppressed.

^b^ Odds ratio for mortality in unknown group could not be estimated owing to zero count.

## Discussion

To our knowledge, this cohort study is the largest single-database study of women giving birth with COVID-19, and we found that women with COVID had an overall peripartum death rate of 0.1%. The death rates and need for ICU admission and respiratory failure requiring intubation were significantly higher among COVID-19 women. Our findings are similar to those by Jering et al,^2^ who reported a 0.14% (9 of 6380 women) death rate for women giving birth with COVID-19 vs 0.005% (20 of 400 066 women) among women without COVID-19.^2^ These data also align with the international living meta-analysis by Allotey et al,^6^ which found increased admission to ICU and increased need for invasive ventilation for pregnant and recently pregnant women with COVID-19 compared with a cohort of nonpregnant women of reproductive age.

Our study also found that women who had COVID-19 were more likely to be Black or Hispanic compared with women without COVID-19. This information is critically important given the ongoing issues surrounding health care disparities and race. When considering mortality during childbirth, it is important to understand that racial disparities have been well established preceding the COVID-19 pandemic; however, they have likely been augmented by the pandemic.^8,9,10^ In fact, previous data from the Centers for Disease Control and Prevention have demonstrated that Black and American Indian or Alaskan Native mothers in the US are 2- to 3-fold more likely to die from pregnancy-related causes. Of note, pregnancy-related mortality rate in the US has been increasing over the last 10 years and is now approximately 0.017%. The mortality rate is approximately 0.044% for Black mothers.^9^ While the reasons behind this difference are complex, much research has supported the role of health disparities, social and structural determinants of health, discrimination, health care access and quality, and disproportionate burden of underlying comorbidities.^11^ Each of these factors has been heightened during the COVID-19 pandemic, and therefore further study on the association of COVID-19 with outcomes among mothers from these communities is necessary.

 Our subset analysis based on gestational age found that women with COVID-19 were more likely to deliver at less than 37 weeks than women who did not have COVID-19. Our findings were consistent with a study by Villar et al^5^ that found higher rates of preterm birth in a multinational cohort study, as well as with Jering and colleagues,^2^ who reported that COVID-19 was associated with higher odds of preterm birth. This is in contrast to a study by Adhikari and colleagues^12^ that reported no difference in preterm birth between women with COVID-19 vs those without. Our findings suggest that additional research is warranted to understand the physiological mechanism of COVID-19 in preterm birth.

### Limitations

There are several limitations to this study, including those inherent to retrospective administrative database studies, including missing data, misclassification, and potential coding inaccuracy. The CDB/RM database is limited to in-hospital mortality and does not contain follow-up data; thus, reported mortality likely underestimates the true mortality. Other limitations include the inability to distinguish patients with COVID-19 who were symptomatic and who were admitted for management of their symptoms versus patients who were asymptomatic and had test results positive for SARS-CoV-2 infection when they were admitted for their birth.

## Conclusions

This cohort study found that women with COVID-19 who were giving birth had a statistically significantly higher mortality rate of 0.13% compared with women without COVID-19. The need for ICU admission and respiratory failure requiring intubation were statistically significantly higher among women with COVID-19. Future research is needed to further understand the pathophysiology of COVID-19 during pregnancy and to better characterize the long-term sequelae.
